# Glomerular Organization of the Antennal Lobes of the Diamondback Moth, *Plutella xylostella* L.

**DOI:** 10.3389/fnana.2019.00004

**Published:** 2019-02-05

**Authors:** Xizhong Yan, Zhiyu Wang, Jiaoxin Xie, Caiping Deng, Xuejun Sun, Chi Hao

**Affiliations:** ^1^Department of Entomology, Agricultural College, Shanxi Agricultural University, Taigu, China; ^2^Department of Entomology, Forestry College, Shanxi Agricultural University, Taigu, China; ^3^Department of Experimental Oncology, Cross Cancer Institute, University of Alberta, Edmonton, AB, Canada

**Keywords:** antennal lobe, glomeruli, olfactory system, confocal microscopy, antennal backfill, anatomical reconstruction, *Plutella xylostella*

## Abstract

The antennal lobe of the moth brain is the primary olfactory center processing information concerning pheromones and plant odors. *Plutella xylostella* is a major worldwide pest of cruciferous vegetables and its behavior is highly dependent on their olfactory system. However, detailed knowledge of the anatomy and function of the *P. xylostella* olfactory system remains limited. In the present study, we present the 3-Dimentional (3-D) map of the antennal lobe of *P. xylostella*, based on confocal microscopic analysis of glomerular segmentation and Neurobiotin backfills of Olfactory Receptor Neurons (ORNs). We identified 74–76 ordinary glomeruli and a macroglomerular complex (MGC) situated at the entrance of the antennal nerve in males. The MGC contained three glomeruli. The volumes of glomeruli in males ranged from 305.83 ± 129.53 to 25440.00 ± 1377.67 μm^3^. In females, 74–77 glomeruli were found, with the largest glomerulus ELG being situated at the entrance of the antennal nerve. The volumes of glomeruli in females ranged from 802.17 ± 95.68 to 8142.17 ± 509.46 μm^3^. Sexual dimorphism was observed in anomalous supernumerary, anomalous missing, shape, size, and array of several of the identified glomeruli in both sexes. All glomeruli, except one in the antennal lobe (AL), received projections of antennal ORNs. The glomeruli PV1 in both sexes received input from the labial palp nerve and was assumed as the labial pit organ glomerulus (LPOG). These results provide a foundation for better understanding of coding mechanisms of odors in this important pest insect.

## Introduction

In insects, the olfactory system plays a highly important role in detecting odorants involved in sexual communication, social integration, host recognition, and escaping from enemies over a distance (Lopes et al., 2002; Gill et al., 2013; Sun et al., 2014; Xu et al., 2016). The antennae are the primary olfactory organ and bear several types of sensilla (Galizia and Rossler, 2010; Yan et al., 2014, 2017a). The most prominent sensillum types have olfactory functions and contain olfactory receptor neurons (ORNs), which send projections directly to the antennal lobe (AL), the primary olfactory center of the insect brain.

Typically, the insect AL is composed of many spheroidal neuropilar units, called glomeruli that house synaptic contacts between receptor axons and AL interneurons (Hansson and Stensmyr, 2011). The arrangement, size, and number of AL glomeruli are species-specific, allowing the identification of individual glomeruli according to the size, shape, and its position. The number of glomeruli in the AL varies from fewer than 15 glomeruli in *Scaphoideus titanus* (Rossi Stacconi et al., 2014) to more than 1,000 in locusts (Rospars, 1988). Most insect species studied to date have 40–160 glomeruli arranged in a single or double layer (Rospars, 1983; Hansson and Anton, 2000). In many insect species, sexual dimorphism with respect to specific glomeruli is observed, e.g., the AL of male Lepidoptera contains enlarged glomeruli that form the macroglomerular complex (MGC) at the entrance of the antennal nerve into the Al in *Mamestra brassicae* (Rospars, 1983) and *Trichoplusia ni* (Todd et al., 1992). All ORNs that express the same specific odorant receptor converge onto the same glomerulus (Vosshall and Wong, 2000). Odor representation from ORNs to projection neurons (PNs) is generally conserved (Seki et al., 2017). Different PN classes target dendrites to distinct olfactory glomeruli, whereas PNs of the same class exhibit indistinguishable anatomical and physiological properties (Li et al., 2017). In the *Drosophila* olfactory circuit, 50 ORN classes and 50 PN classes form synaptic connections in 50 glomerular compartments in the antennal lobe, each of which represents a discrete olfactory information-processing channel (Wu et al., 2017). The one ORN class to one glomerulus and to one PN class relationship in the *Drosophila* olfactory system is likely an extreme situation as, in other species, this may vary as the glomeruli numbers vary dramatically. In male moths, the MGC receives and processes information regarding the female sex pheromone, calcium imaging in *Heliothis virescens* confirms that sex pheromone responses are restricted to the male-specific MGC, plant odors to ordinary glomeruli (Galizia et al., 2000), and sex pheromone-specific receptor neurons arborize in the MGC (Hansson et al., 1992; Ochieng et al., 1995).

The diamondback moth (DBM; *Plutella xylostella*; Lepidoptera: Plutellidae) is a major worldwide pest of cruciferous vegetables. Because of the intensive and extensive application of insecticides for the control of *P. xylostella*, the species has become one of the most resistant insect pests in the world (Sarfraz et al., 2005; Furlong et al., 2013; Wang et al., 2013; Li et al., 2016). This resistance has prompted increasing efforts to identify new, natural approaches to control this insect. One of these approaches focuses on understanding the plant chemistry that plays a major role in the moth acceptance or rejection of host plants. However, relatively little is known about the olfactory pathways in the central nervous system of this insect. To better understand the general organization and the functional significance of AL glomeruli in odor processing, we generated the first digital 3-D reconstruction of the AL in *P. xylostella* through Confocal Laser Scanning Microscopy (CLSM) reconstruction of the AL and backfilling of the antennal neurons. The atlas will serve as a template for future analysis of physiological responses in morphologically identified glomeruli in this pest insect.

## Materials and Methods

### Insects

*Plutella xylostella* were reared at the temperature of 25°C, relative humidity of 75% and photoperiod of (L:D = 14:10) in the Insect Neuroethology & Sensory Biology laboratory, Shanxi Agricultural University. Pupae were individually segregated into test tubes according to sex, and recently emerged (1–2-days-old) female and male adults of *P. xylostella* were used in this study.

### Palp and Antennal Backfills

Six male and six female moths were anesthetized with carbon dioxide and immobilized using double-sided adhesive tape, and the ventral part of the head was exposed. The antennae or the palp of one side of the moth were cut near the base, and the remainder of the segments were inserted for 30 min in an appropriately sized micropipette tip filled with 2% Neurobiotin (Neurobiotin Tracer, Vector Laboratories, Burlingame, USA) solution. After the micropipette was removed, the preparations were then incubated at 4°C for 2 h in a humidified and dark box for diffusion of the dye. Subsequently, the brains were dissected out in 4% paraformaldehyde in phosphate buffer (PB, 0.1 M, pH 7.2) under a dissecting microscope, and the isolated brains were fixed in the same fixative at 4°C for 1-2 days for batch processing. After rinsing three times with 0.1% Triton X-100 in 0.1 M PB, the brains were incubated in 2 μg/ml Alexa-488 Streptavidin (Molecular Probes, Life Technologies, USA) solution in PBS for 1-2 days and then in 10 μg/ml Propidium Iodide (Sigma) solution for 30 min at room temperature. The brains were dehydrated in an ascending ethanol series of 30, 50, 75, 95, and 100% alcohol and then embedded in DPX (Sigma) after a brief xylene transition.

### Confocal Laser Scanning Microscopy

Twelve whole-mounts of brains were imaged with a Zeiss LSM 710 confocal laser scanning microscope equipped with multiple laser lines that permitted the visualization of structures labeled with Alexa-488 and labeling of Propidium iodide used in this study. CLSM imaging was conducted using 488-nm excitation wavelength for the Alexa-488 Streptavidin labeling, and the Alexa-488 signal was collected between 490 and 560 nm. The propidium iodide signals, which label the nucleus of neurons, were excited with a 561 nm laser line and collected between 567 and 596 nm. For overview scanning of the whole brain and detailed scanning of the antennal lobe, a 40× (NA1.3) oil objective was used with a sampling rate matching the Nyquist sampling rate with a pinhole setting of 1 Airy Unit. Image data were captured as serial stacks (pixel dwelling time of 0.39 μs, pinhole 1 AU, step size 0.4–0.45 μm). Generally, each brain requires an image stack of 300–500 slices (depending on the mounting orientation of the brain) of images with 2048 × 2018 pixels [with a pixel dimension of 0.104 × 0.104 × 0.4 μm (x, y, z)].

### 3-D Reconstructions and Identification of Glomeruli

**Figure 1:**
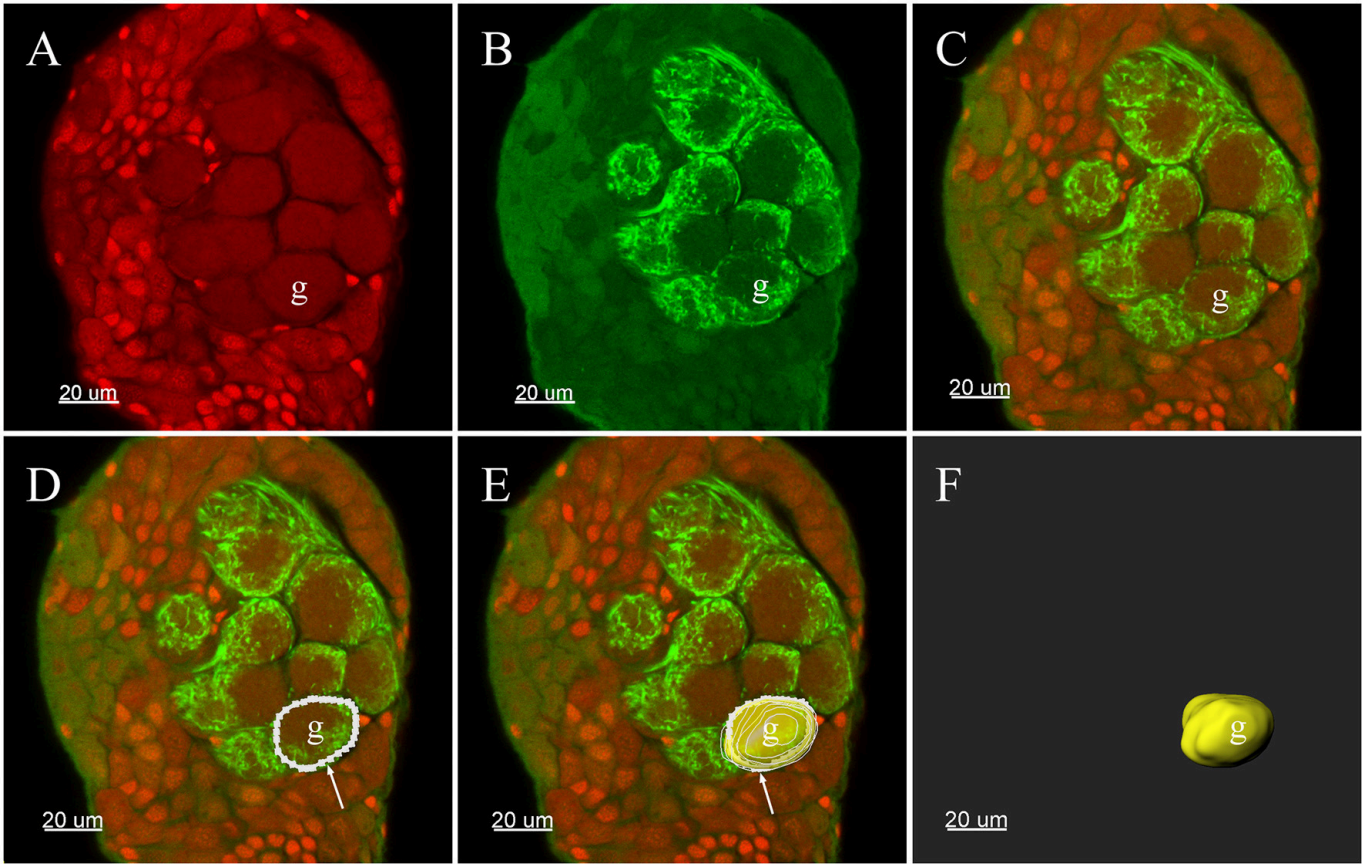
Three-D reconstructions of a glomerulus in the antennal lobe of *Plutella xylostella* demonstrating the procedures for the 3-D reconstruction. **(A)** One raw confocal image of the antennal lobe with Propidium Iodide labeling the cell nucleus. **(B)** Neurobiotin™ backfilling of the antennal sensory innervations, green indicates signal from the Neurobiotin of the projections of ORNs. **(C)** Propidium Iodide and Neurobiotin stainings merged together from **(A)** and **(B)**. **(D)** Tracing the outline of one glomerulus in a single plane was carried out manually in Imaris. **(E)** After repeating the tracing of other layers of the glomerulus, the glomerulus was surface rendered (yellow) with the outlines (white). **(F)** The surface render of one glomerulus. The arrow indicates outline of a glomerulus. g, Glomerulus.

All confocal image stacks were viewed and processed with the 3-D reconstruction software Imaris (vers: 8.4.0, Bitplane, Zurich, Switzerland). Glomeruli in the ALs were entirely demarcated by manually tracing the outline of each glomerulus in every other section of the stack file. The outlines of the glomeruli could be easily defined by a combination of many characteristics in the images: auto-fluorescence of the antennal lobe structure, propidium iodide-labeled glia cells that usually surround the glomeruli (Figures 1A,C) (Yan et al., 2017b) and signals from the Neurobiotin backfills of the ORNs (Figures 1B,C). Such manually delineated lines were subsequently used to generate surface rendered glomeruli structure, and the software generated the volume as well as sphericity of each individual glomerulus (Figures 1D–F). Two analysts independently analyzed each data set to reduce possible errors and bias. Glomeruli were identified according to their location, shape, and size. The glomerular nomenclature used was adopted from previous publications of other groups (Ignell et al., 2005; Solari et al., 2016). Namely, each glomerulus was marked by one or two capital letters indicating the general position: anterior (A), posterior (P), ventral (V), dorsal (D), lateral (L), medial (M), and central (C). Letters were followed by numbers to indicate glomerulus presentation order from the most anterior to the most posterior in the same region. Additionally, the glomeruli were identified using several structural landmarks such as (1) general contour structure of the AL; (2) the entrance of the antennal nerves; (3) the brain orientations, and (4) specific, easily identifiable glomeruli such as putative MGC, enlarged glomeruli in the female, ELG, PV1, etc. The same nomenclature structure was used for both the male and female glomeruli. Although we did not systematically compare the glomeruli from the male and female AL, every attempt was made to have glomeruli in both sexes in similar positions and sizes to have comparable glomeruli annotation. This visualization based matching presented some inherited limitations that the same glomeruli name may not present the homologous glomeruli. Every glomerulus was assigned with a random color for the purpose of visualization. The MGC received served as an essential orientation landmark for the male AL. Final output data were stored in TIFF format and annotated using Photoshop CS software (Adobe, San Jose, CA, USA).

### Statistical Analyses

The following parameters were measured: total number of glomeruli and volume of glomeruli of the AL. The volume of each individual glomerulus was calculated with the SPSS statistical software package (ver. 13.0, SPSS incorporated, Chicago, Illinois), and values are presented as the mean ± SD.

## Results

### Three-Dimensional Reconstruction of the AL

**Figure 2:**
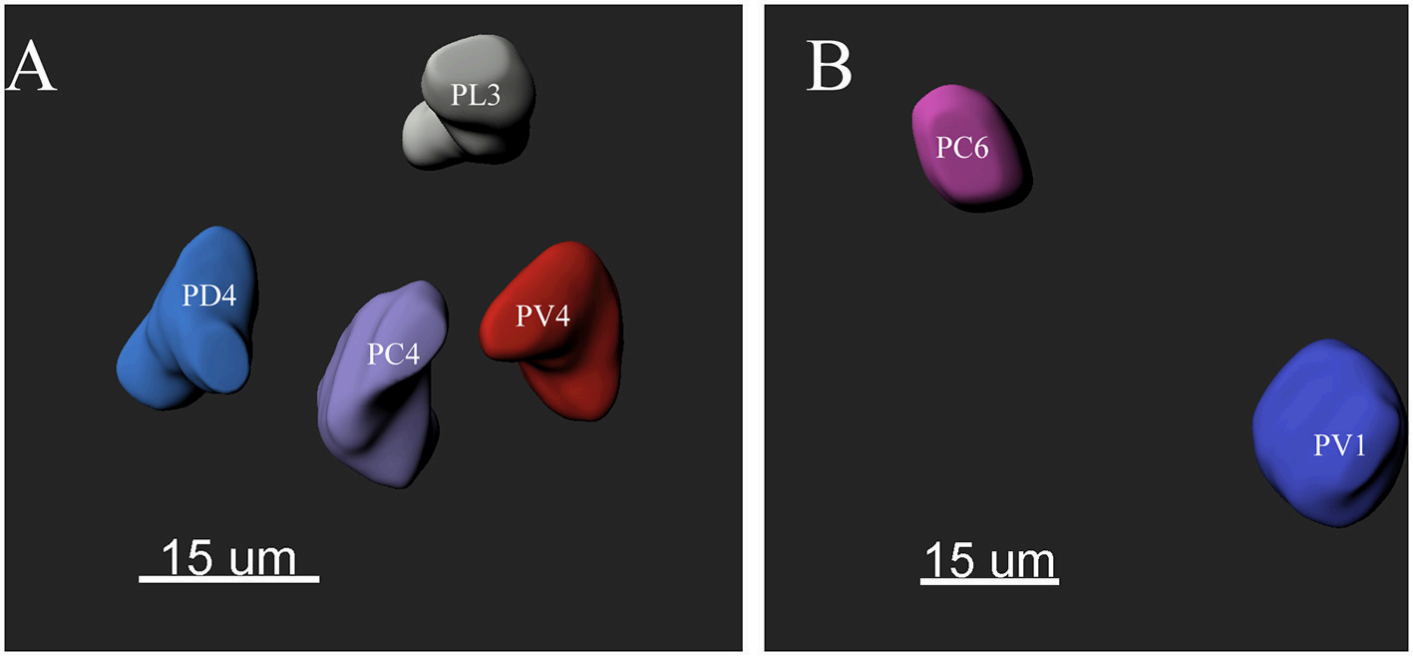
The morphology of the glomeruli in a female antennal lobe of *P. xylostella*. **(A)** Irregular glomeruli (sphericity: PV4: 0.852; PC4: 0.842; PL3: 0.834 and PD4: 0.832). **(B)** Regular glomeruli (sphericity: PC6 0.946 and PV1: 0.950).

**Figure 3:**
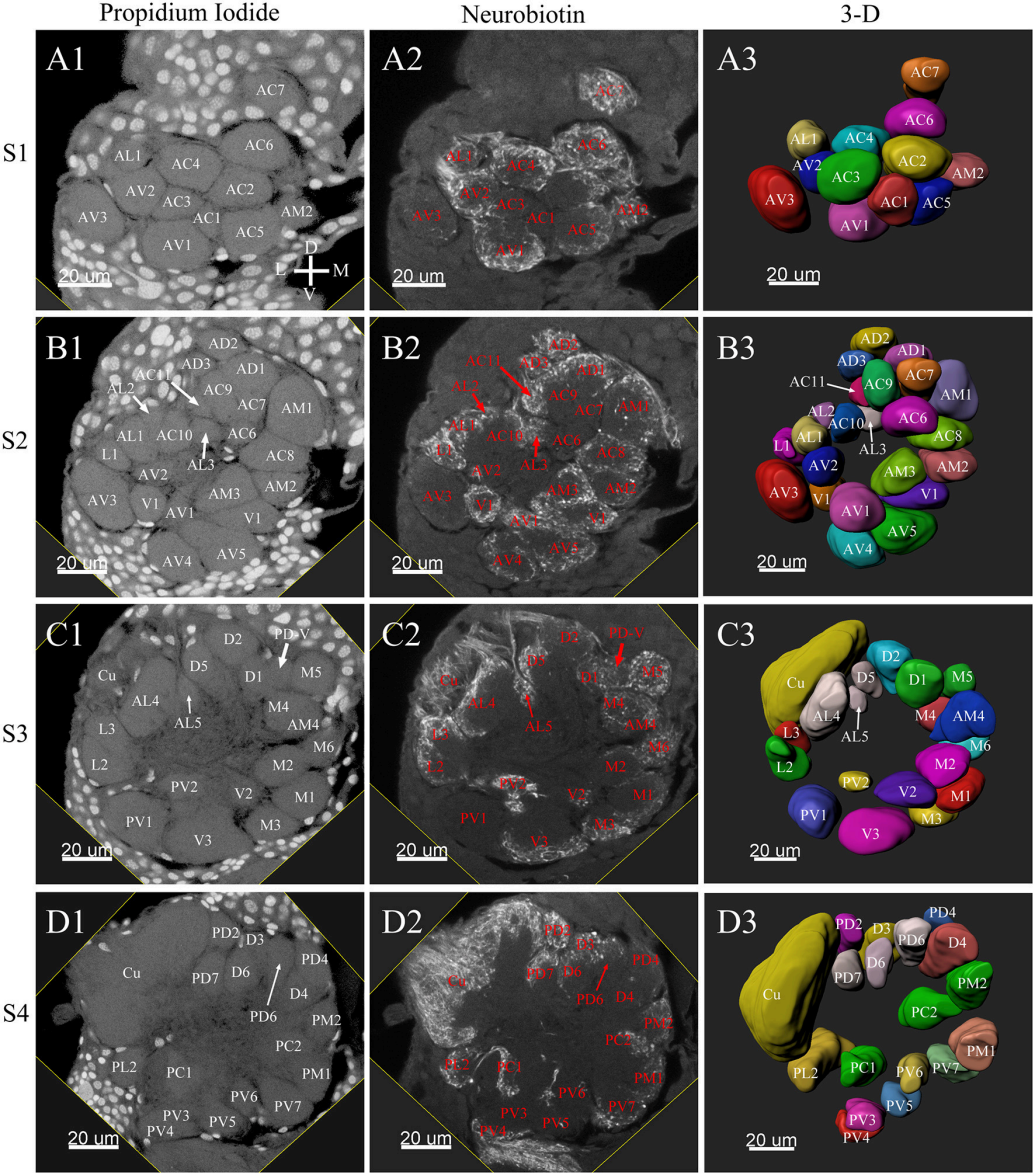
Frontal glomeruli reconstructions of a male antennal lobe. For clarity, the entire antennal lobe was separated into 7 consecutive arbitrarily reconstructed series (S1-S7). This panel shows the front 4 series (last 3 series will be demonstrated in Figure 4): **(A1–A3)** The first level, S1, where was the most anterior layer. The **(B1–B3)** shows the second level, S2. The **(C1–C3)** depicts the third level, S3. The **(D1–D3)** shows the fourth level, S4. **(A1–D1)** Series of frontal confocal sections of Propidium Iodide labels; **(A2–D2)** Neurobiotin backfilling of the antennal sensory innervations. **(A3–D3)** Frontal view of 3-D reconstructions of the antennal lobe. Each identified glomerulus was named according to the monocultures as described in the text and numbered, from the most anterior to the most posterior in each layer. Additionally, different colors were randomly assigned to each glomerulus. Cu, cumulus; D, dorsal; L, lateral; M, medial; V, ventral.

**Figure 4:**
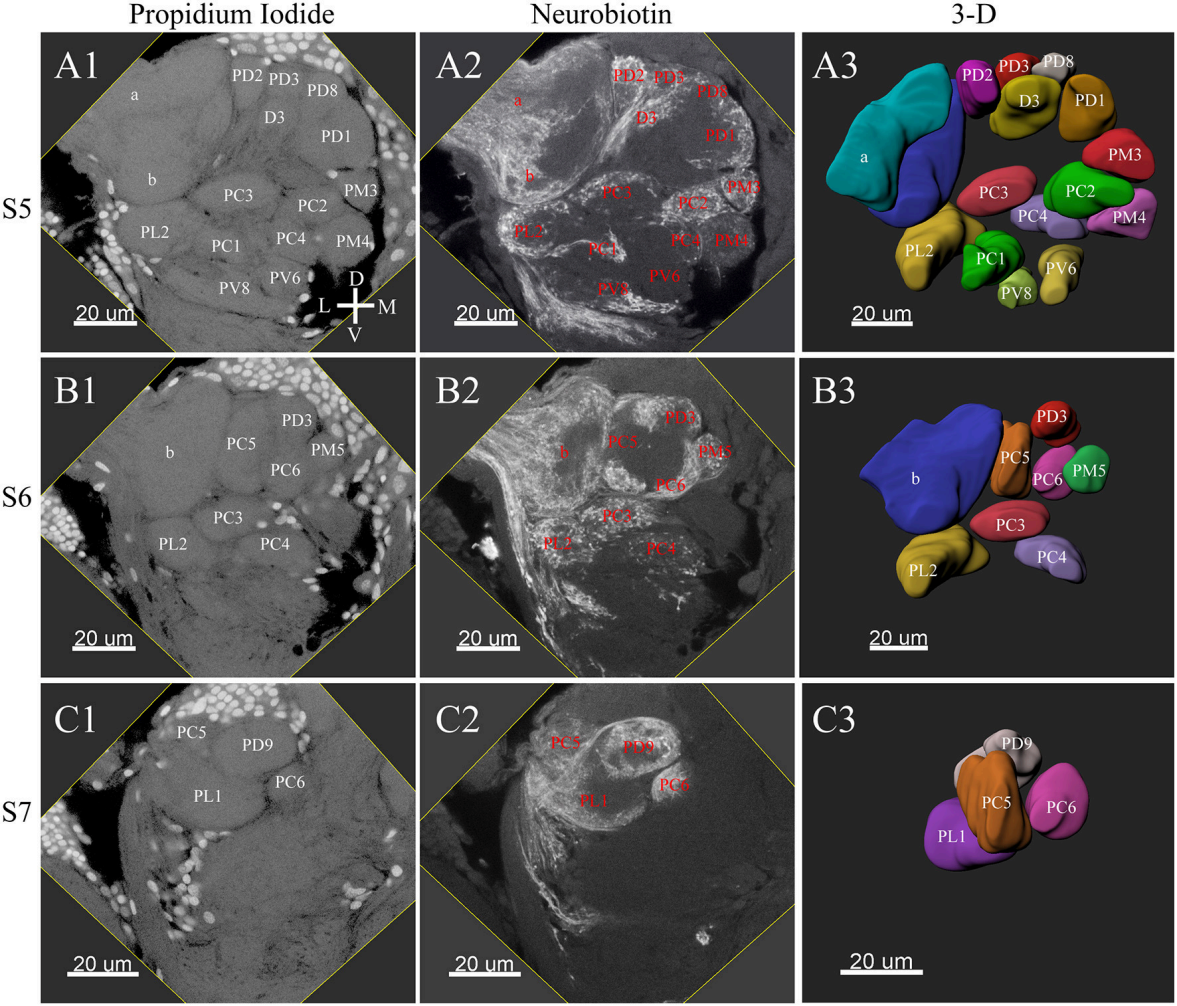
Posterior glomeruli reconstructions of a male antennal lobe, series S5-7. Row **(A1–A4)** shows the fifth level, S5. Row **(B1–B4)** demonstrates the sixth level, S6. The row **(C1–C4)** shows the most posterior glomeruli, the seventh level, S7. **(A1–C1)** Series of frontal confocal sections of Propidium Iodide. **(A2–C2)** Neurobiotin backfilling of antennal sensory innervations. **(A3–C3)** Frontal views 3-D reconstructions. Each identified glomerulus was named and numbered from the most anterior to the most posterior as described in text. D, dorsal; L, lateral; M, medial; V, ventral.

**Figure 5:**
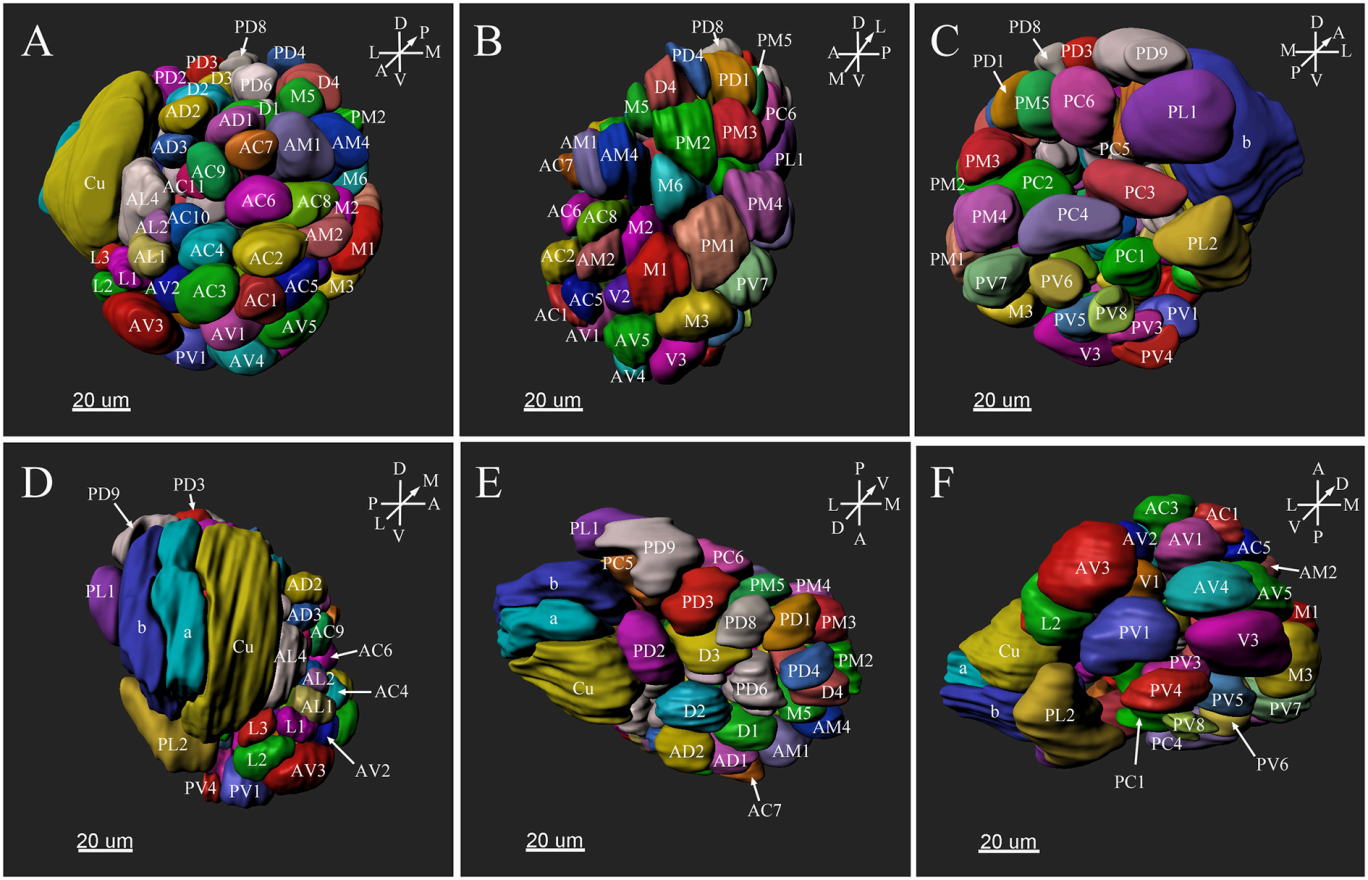
Three-dimensional reconstruction of the entire antennal lobe of a male *P. xylostella*. **(A)** Frontal view. **(B)** Medial view. **(C)** Posterior view. **(D)** Lateral view. **(E)** Dorsal view. **(F)** Ventral view. A, anterior; D, dorsal; L, lateral; M, medial; P, posterior; V, ventral.

**Figure 6:**
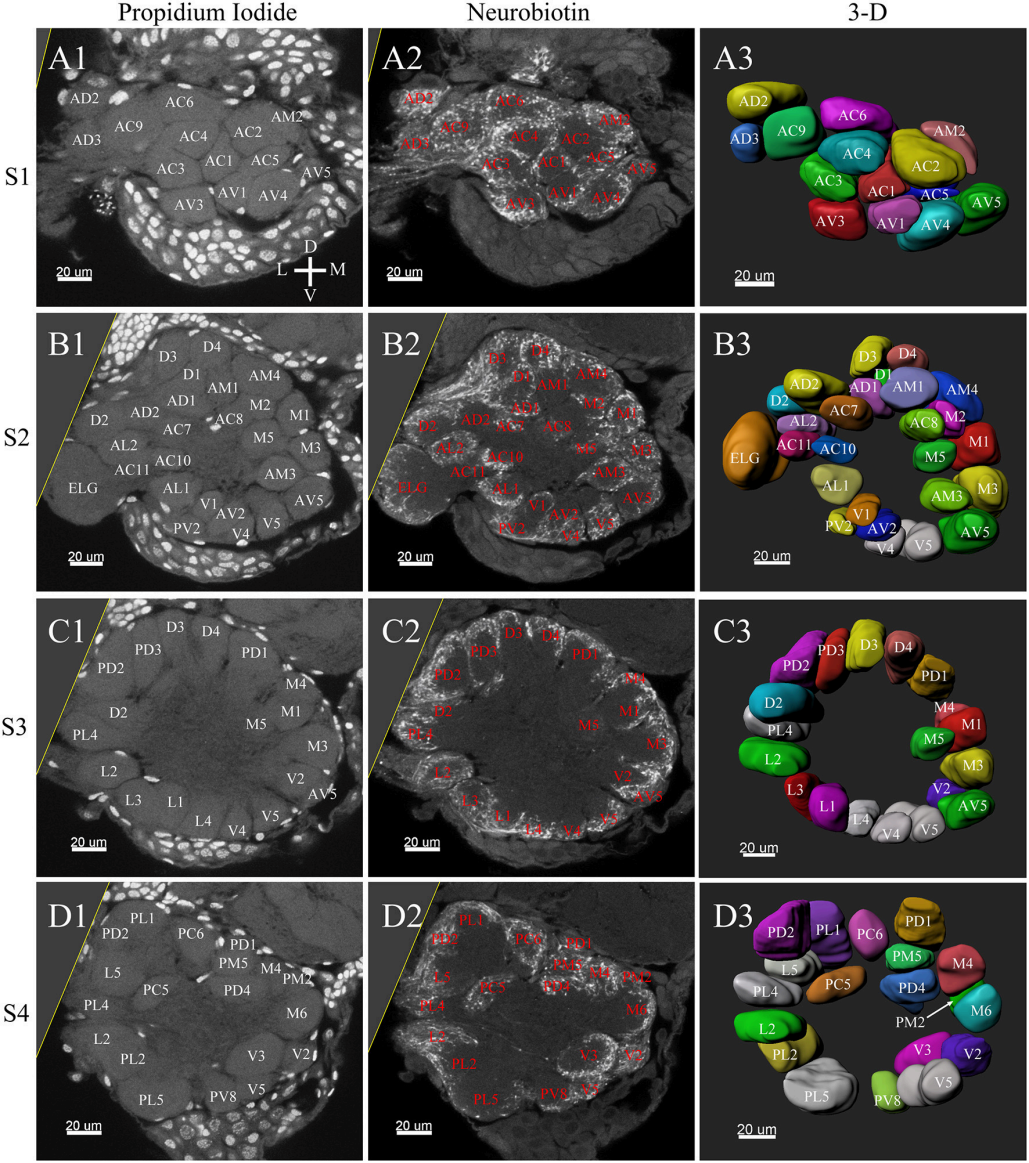
Frontal glomeruli reconstructions of a female antennal lobe. For clarity, the entire antennal lobe was separated from front to posterior into series of 7 layers (S1–S7). The glomeruli from the front 4 layers (S1–S4) are shown in this panel. **(A1–D1)** Confocal sections of Propidium Iodide labeling. **(A2–D2)** Neurobiotin back filling labeling of antennal sensory innervations. **(A3–D3)** Frontal views of the 3-D reconstructions of the antennal lobe. The homologous glomeruli were named and color-coded in the same manner as in the male reconstruction. D, dorsal; L, lateral; M, medial; V, ventral.

**Figure 7:**
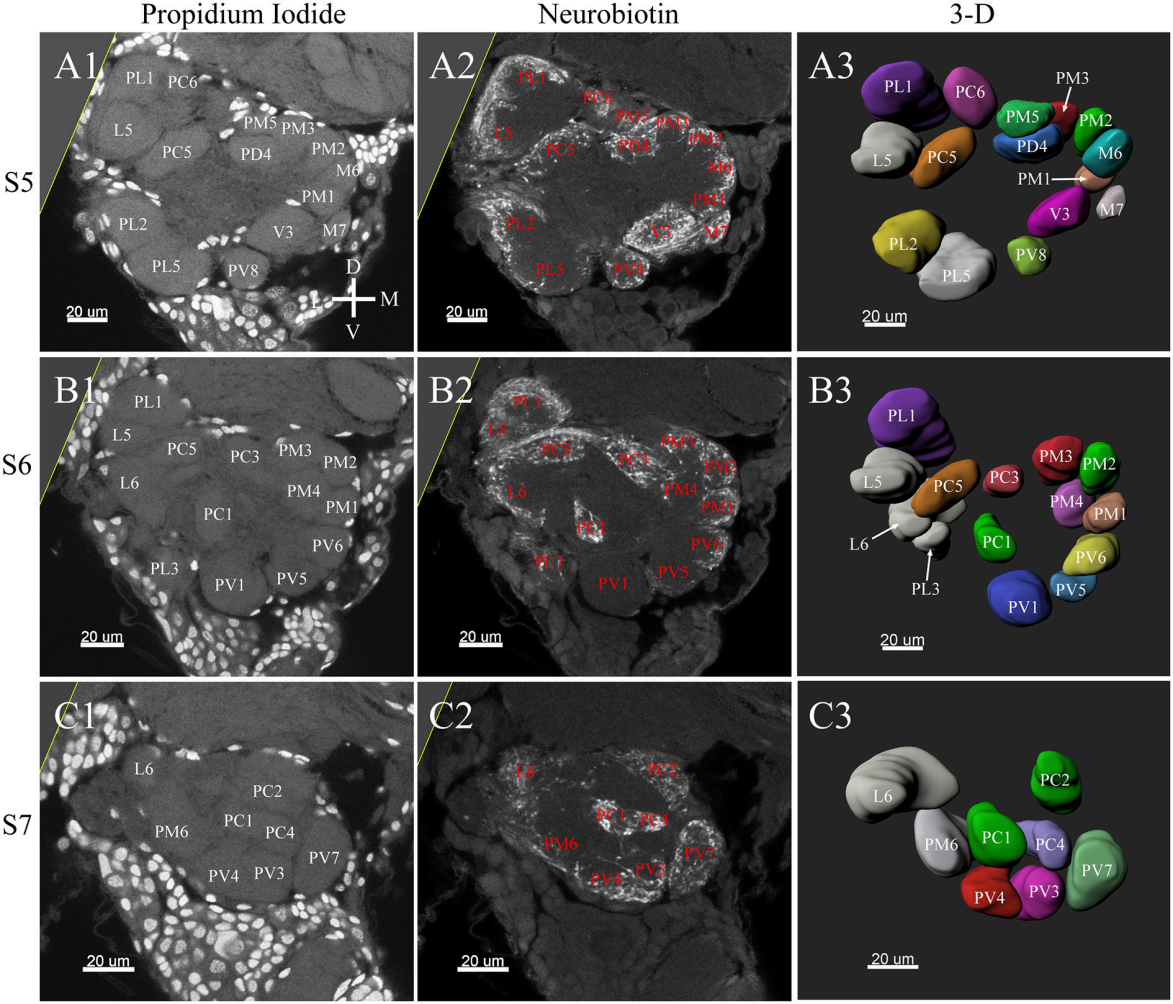
Posterior glomeruli reconstructions of a female antennal lobe, series S5–7. **(A1–C1)** Series of frontal confocal sections of Propidium Iodide labeling. **(A2–C2)** Neurobiotin labeling of the antennal sensory innervations. **(A3–C3)** Front views of the 3-D reconstructions of the antennal lobe. Each identified glomerulus was named and as described before. D, dorsal; L, lateral; M, medial; V, ventral.

**Figure 8:**
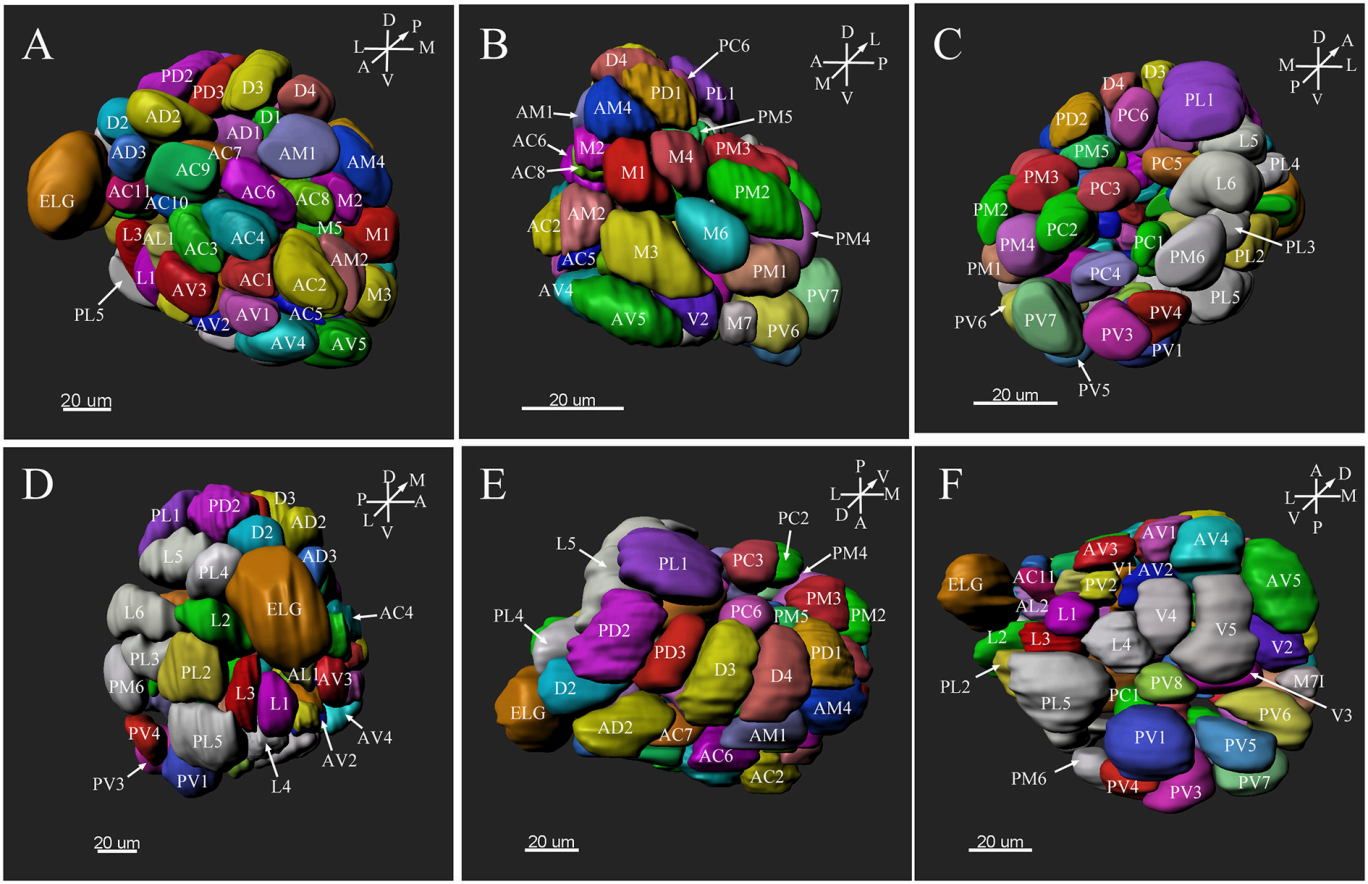
Three-dimension reconstruction of the glomeruli from the entire antennal lobe of a female *P. xylostella*. **(A)** Frontal view. **(B)** Medial view. **(C)** Posterior view. **(D)** Lateral view. **(E)** Dorsal view. **(F)** Ventral view. A, anterior; D, dorsal; L, lateral; M, medial; P, posterior; V, ventral.

The ALs of *P. xylostella* were mostly sphere-shaped structures located in the front most part of the brain. Numerous glomeruli were found in the AL (Supplementary Videos 1–3). Generally, the majority of the glomeruli were spherical or elliptical in shape. The sphericities (a measure of how round a structure is) of the glomeruli were in the range of 0.7–0.96 (while a perfect sphere is 1). Only few glomeruli per antennal lobe displayed irregularities with a lower sphericity (Figure 2). The glomeruli were mostly located at the periphery of the lobe in a single layer and generally, the anterior glomeruli were more densely packed than the posterior glomeruli (Figures 3–**8**). Most glomeruli were uniform in shape, size, and relative position, based on the comparison of different ALs and therefore could be identified and recognized individually. Only glomeruli four and five displayed some anomalies (missing or extra) (**Table 3**). The glomeruli were named and color-coded as descripted previously. Each identified glomerulus was named and numbered in the order in which the layers appear from the most anterior to the most posterior.

Tracing neurons from the antenna reliably labeled to the ipsilateral AL, and projections of ORNs formed densely-packed regions identifiable as glomeruli (Figures 3A2–D2, 4A2–C2, **6A2–D2**, **7A2–C2**).

### Male ALs

A putative Macro-Glomerular Complex (MGC) structure and 74–76 ordinary glomeruli were found in the six male ALs studied (Table 1; Figures 3–5; Supplementary Video 2).

**Table 1:** Volumes of glomeruli (mean ± SE) in the antennal lobe (AL) of male *Plutella xylostella*.

**Glomerulus**	**Volume (μm^**3**^)**	***n***	**Glomerulus**	**Volume (μm^**3**^)**	***n***	**Glomerulus**	**Volume (μm^**3**^)**	***n***
AC1	1455.83 ± 263.79	6	AV5	3811.17 ± 249.27	6	PD9	4705.33 ± 846.81	6
AC2	2302.67 ± 535.07	6	L1	1190.67 ± 172.22	6	PL1	5489.33 ± 956.17	6
AC3	1785.17 ± 387.84	6	L2	3303.83 ± 898.02	6	PL2	9166.50 ± 1631.38	6
AC4	1697.83 ± 147.12	6	L3	2053.17 ± 952.25	6	PV1	4573.00 ± 658.85	6
AC5	1238.17 ± 87.83	6	M1	2870.00 ± 721.05	6	PV2	926.50 ± 101.11	2
AC6	1572.17 ± 248.01	6	M2	2425.33 ± 428.86	6	PV3	3616.50 ± 486.37	6
AC7	1238.67 ± 178.11	6	M3	4030.83 ± 209.03	6	PV4	1371.17 ± 274.65	6
AC8	1957.67 ± 338.50	6	M4	1779.67 ± 310.97	6	PV5	1621.83 ± 137.32	6
AC9	1669.33 ± 263.82	6	M5	1043.40 ± 159.47	5	PV6	3018.67 ± 504.12	3
AC10	919.00 ± 90.10	6	M6	2342.17 ± 160.17	6	PV7	3178.00 ± 457.11	6
AC11	983.00 ± 179.51	6	D1	1620.17 ± 305.26	6	PV8	996.50 ± 218.69	6
AM1	2472.33 ± 215.57	6	D2	2085.17 ± 430.69	6	PM1	2894.50 ± 684.68	6
AM2	1671.67 ± 289.38	6	D3	2218.17 ± 412.21	6	PM2	2529.00 ± 553.86	6
AM3	1728.667 ± 216.59	6	D4	2243.83 ± 306.97	6	PM3	2346.83 ± 451.21	6
AM4	1572.50 ± 238.83	6	D5	777.67 ± 252.99	6	PM4	2456.67 ± 291.34	6
AL1	1269.17 ± 325.48	6	D6	1575.50 ± 189.71	6	PM5	1867.00 ± 99.62	6
AL2	305.83 ± 129.53	6	V1	1053.00 ± 578.49	6	PC1	2192.33 ± 622.91	6
AL3	1239.17 ± 159.30	6	V2	2584.83 ± 431.54	6	PC2	2704.83 ± 459.46	6
AL4	2931.33 ± 156.19	6	V3	3735.00 ± 348.08	4	PC3	3177.33 ± 1084.69	6
AL5	675.83 ± 272.42	6	PD1	2156.17 ± 383.89	6	PC4	3469.50 ± 1377.57	6
AD1	1724.50 ± 234.17	6	PD2	2490.17 ± 219.98	6	PC5	3301.50 ± 1555.46	6
AD2	1799.83 ± 172.00	6	PD3	1978.17 ± 268.26	6	PC6	2547.67 ± 377.95	6
AD3	1102.83 ± 117.33	6	PD4	1987.50 ± 250.18	6	Cu	25440.00 ± 1377.67	6
AV1	2127.67 ± 611.54	6	PD5	1463.17 ± 279.17	6	a	12540.00 ± 968.50	6
AV2	1423.67 ± 282.43	6	PD6	1848.33 ± 213.48	6	b	16640.00 ± 1818.79	6
AV3	2923.33 ± 779.33	6	PD7	1681.33 ± 151.52	6			
AV4	2534.00 ± 432.38	6	PD8	1253.67 ± 124.44	6			

*Underlined texts indicate missing/abnormal glomeruli*.

### The MGC

The putative MGC structure was found close to the entrance of the antennal nerve of male *P. xylostella*. It contained three glomeruli: the cumulus and glomeruli a and b (Figures 3D1–3, 4A1–3, B1–3, 5D). They were clearly separated from the array of ordinary glomeruli, based on their shape, volume, and location. As the most anterior glomerulus of the putative MGC, the cumulus exhibited a cylindrical shape, but the ventral part was flattened (Figure 3C3). The cumulus was the largest glomerulus in the AL, and the volume was 25440.00 ± 1377.67 μm^3^ (Table 1). Glomerulus a of the putative MGC positioned posteriorly from the cumulus, and the volume was 12540.00 ± 968.50 μm^3^. Glomerulus b of the putative MGC positioned posteriorly from glomerulus a and was 16640.00 ± 1818.79 μm^3^ in volume (Figure 5D; Table 1). All three glomeruli in the MGC received projections of ORNs from the antenna (Figures 3D2, 4A2,B2). The cumulus and glomeruli a and b in MGC were regarded as very important landmark glomeruli.

### Ordinary Glomeruli

**Figure 9:**
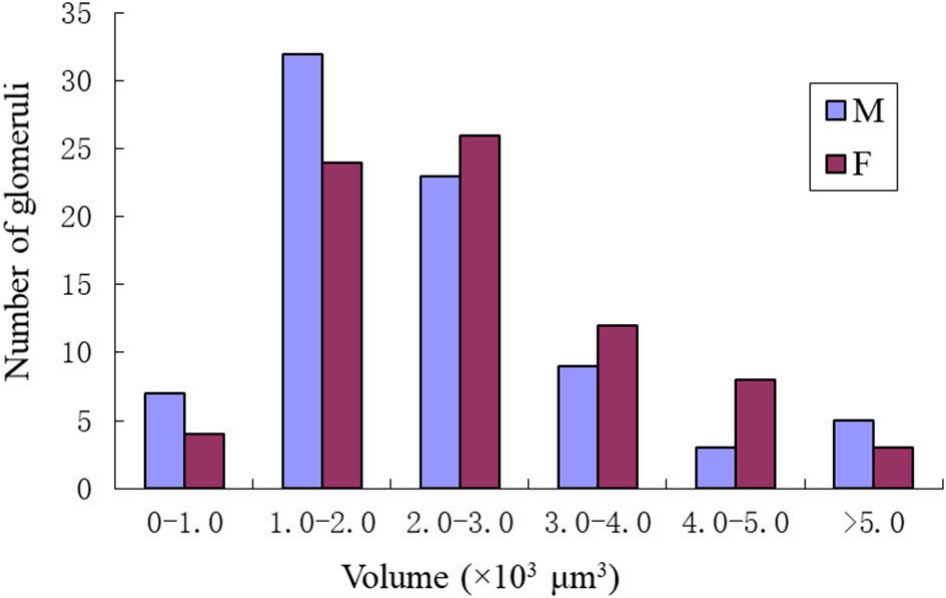
Size distributions of the glomeruli in the male and female of *P. xylostella*. Note the male has the most glomeruli in the volume of 1–2 × 1000 μm^3^ while the female has the most glomeruli with the volume of 2–3 × 1000 μm^3^.

**Figure 10:**
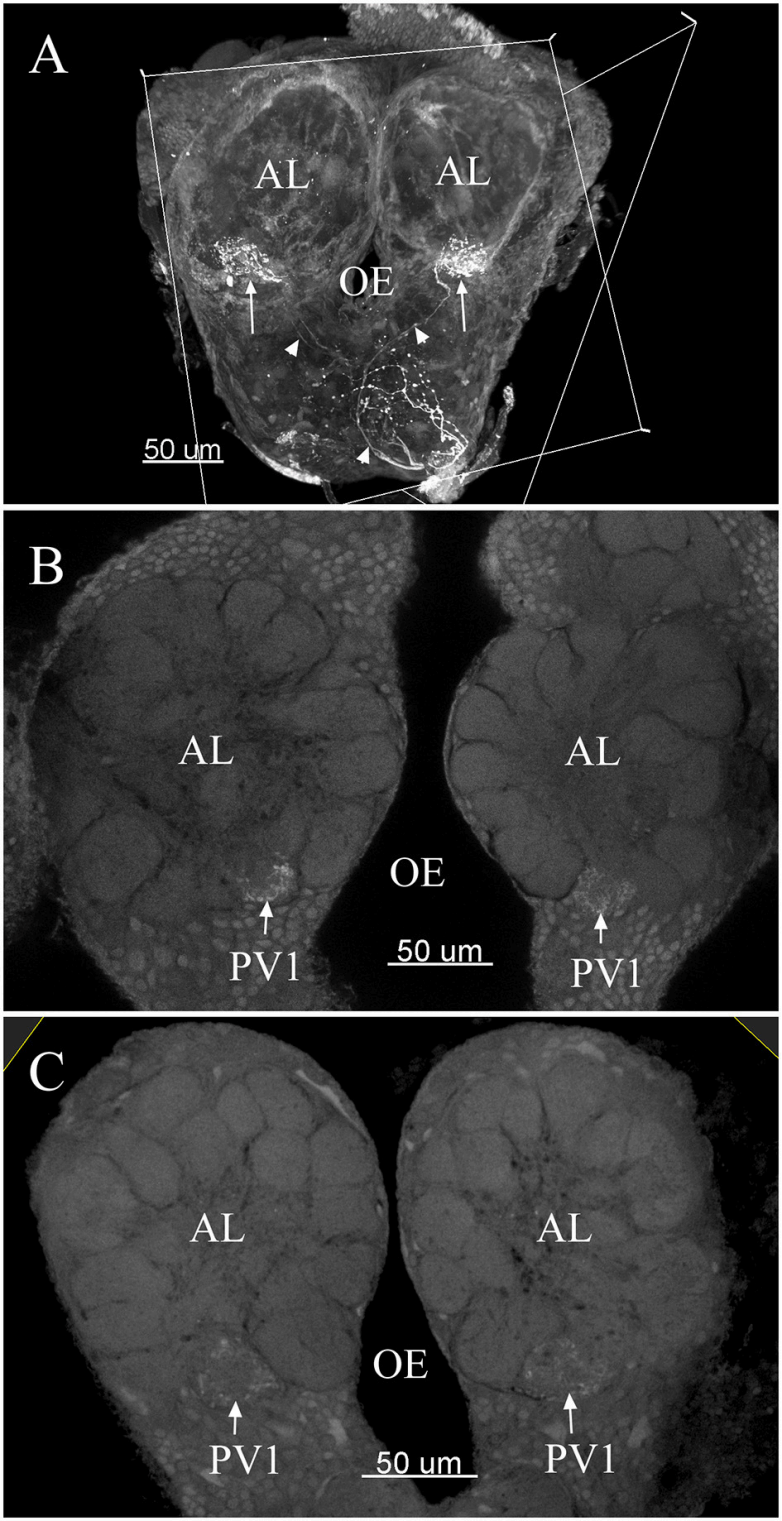
The confocal images of the terminals of sensory neurons originating from the labial palpus projecting bilaterally to the AL. Note the back-fillings were performed on a single side of the brains **(A)**. PV1 ordinary glomerulus in male **(B)** and PV1 in female **(C)**. The arrow indicates nerve terminals project bilaterally into the ordinary glomerulus. The arrowhead indicates nerve fibers. AL, antennal lobe; OE, esophagus.

The maximum number of ordinary glomeruli was 76, which was found in one male AL. M5, V3, PV2 and PV6 were missing in some ALs within our samples; M5 was found in five of the six ALs, V3 was found in four of the six ALs, PV2 was found in two of the six ALs, and PV6 was found in three of the six ALs. The other 72 glomeruli could be systematically identified in all six ALs (**Table 3**). Seven glomeruli in males had volumes smaller than 1000 μm^3^ (**Figure 9**). Glomerulus AL2 was the smallest ordinary glomerulus, with a volume of 305.83 ± 129.53 μm^3^ (Table 1), and this glomerulus was located in the anterior-lateral region of the AL (Figures 3B1–3, 5A). Most glomeruli showed volumes between 1000 and 3000 μm^3^, with 32 glomeruli volumes between 1000 and 2000 μm^3^, and 23 between 2000 and 3000 μm^3^ in males. In males, 17 glomeruli had volumes larger than 3000 μm^3^, including 14 ordinary glomeruli and the 3 glomeruli in the MGC (**Figure 9**). Glomerulus PL2 was the largest ordinary glomerulus, with a volume of 9166.50 ± 1631.38 μm^3^ (Table 1). PL2 neighbored the MGC and was located in the posterior-lateral region of the AL (Figures 3D1–3, 5D). Antennal backfills revealed staining in many, but not in all glomeruli. Glomerulus PV1 was found without ORN branches (Figure 3C2). Back filling of the labial palps with Neurobiotin performed similarly, revealed only one glomerulus (PV1) was innervated by sensory neurons from the labial palps (**Figure 10B**). This glomerulus had a relatively large size (4573.00 ± 658.85 μm^3^) and was located at the ventral side of the ALs (Figures 5A,F; Table 1). It is worth to note that the back filling of the labial palps nerve labels the PV1 of boths side of the brain, therefore, unlike the other glomeruli, PV1 receives information from both labial palps. The glomeruli AL2, PL2 and PV1 were regarded as landmark glomeruli in male ALs.

### Female ALs

In the six female ALs, 74–77 glomeruli were found (Table 2; Supplementary Video 3). Similar to the male, there was a slight variation in glomeruli numbers: 77 glomeruli were found in the ALs of one female. M3 was found in three of the six AL, M6 was found in two of the six ALs, AM4, M5, and PL3 were found in five of the six ALs, respectively. Nevertheless, 72 glomeruli could be systematically identified in all six ALs (Table 3). Four glomeruli in females had volumes smaller than 1000 μm^3^ (**Figure 9**). AD3 was the smallest glomerulus, and the volume was 802.17 ± 95.68 μm^3^ (Table 2), and this glomerulus was located in the anterior-dorsal region of the AL (Figures 6A1–3, **8A**). In females, 24 glomeruli had volumes between 1,000 and 2,000 μm^3^; the volumes for 26 glomeruli were between 2,000 and 3,000 μm^3^; and an additional 23 glomeruli had volumes larger than 3,000 μm^3^ (**Figure 9**). Corresponding to the location of the MGC in the male, the largest glomerulus ELG was located in the anterior-lateral region of the AL and close to the entrance of the antennal nerve (see ELG in Figures 6B1–3, **8A**), and the volume of this glomerulus was 8,142.17 ± 509.46 μm^3^ (Table 2). All the glomeruli received antennal receptor neurons with the exception of PV1 glomerulus (Figure 7B2), which received neurons from the labial palps, similar to the males (**Figure 10C**). The glomerulus was large (4,961.33 ± 705.50 μm^3^) and was located at the ventral side of the AL (Figures 8D,F; Table 2). The glomeruli ELG, AD3, and PV1 were regarded as landmark glomeruli in female ALs.

**Table 2:** Volumes of glomeruli (mean ± SE) in the antennal lobe (AL) of female *Plutella xylostella*.

**Glomerulus**	**Volume (μm^**3**^)**	***n***	**Glomerulus**	**Volume (μm^**3**^)**	***n***	**Glomerulus**	**Volume (μm^**3**^)**	***n***
AC1	1534.83 ± 310.61	6	L2	3047.00 ± 292.99	6	PL2	4005.83 ± 855.69	6
AC2	2566.00 ± 284.95	6	L3	2078.67 ± 286.74	6	PL3	3210.80 ± 506.80	5
AC3	1669.17 ± 230.16	6	L4	2593.83 ± 440.55	6	PL4	2337.33 ± 472.86	6
AC4	1998.33 ± 263.29	6	L5	4765.83 ± 858.63	6	PL5	6444.83 ± 853.09	6
AC5	1897.50 ± 189.55	6	L6	4620.17 ± 1233.26	6	PV1	4961.33 ± 705.50	6
AC6	1978.33 ± 298.50	6	M1	3321.17 ± 658.54	6	PV2	831.17 ± 374.59	6
AC7	1316.17 ± 204.48	6	M2	1403.17 ± 139.45	6	PV3	2155.00 ± 275.59	6
AC8	1314.83 ± 176.87	6	M3	3897.33 ± 639.76	3	PV4	1762.33 ± 199.61	6
AC9	1751.83 ± 237.46	6	M4	2038.17 ± 350.07	6	PV5	2567.00 ± 234.51	6
AC10	992.83 ± 474.11	6	M5	1498.20 ± 290.72	5	PV6	3039.67 ± 180.81	6
AC11	996.33 ± 108.07	6	M6	2221.50 ± 123.74	2	PV7	3650.83 ± 358.35	6
AM1	2248.00 ± 364.24	6	M7	1090.00 ± 830.53	6	PV8	1564.33 ± 293.87	6
AM2	1776.83 ± 262.43	6	D1	1026.50 ± 91.87	6	PM1	2074.67 ± 347.31	6
AM3	1690.83 ± 919.16	6	D2	2480.83 ± 441.53	6	PM2	2556.00 ± 393.95	6
*AM4*	3627.80 ± 340.08	5	D3	2581.83 ± 446.73	6	PM3	2316.83 ± 314.58	6
AL1	1941.50 ± 331.14	6	D4	2939.67 ± 168.05	6	PM4	2938.00 ± 374.46	6
AL2	1580.33 ± 212.61	6	V1	1146.50 ± 411.47	6	PM5	2123.00 ± 576.27	6
AD1	2077.17 ± 395.49	6	V2	2794.83 ± 370.09	6	PM6	3524.33 ± 435.40	6
AD2	1920.17 ± 485.95	6	V3	2659.67 ± 417.20	6	PC1	2265.83 ± 288.42	6
AD3	802.17 ± 95.68	6	V4	1991.50 ± 434.75	6	PC2	2825.17 ± 508.72	6
AV1	1545.83 ± 239.05	6	V5	4018.33 ± 848.20	6	PC3	3009.67 ± 449.75	6
AV2	1534.67 ± 339.92	6	PD1	2535.50 ± 387.61	6	PC4	2648.00 ± 481.32	6
AV3	3360.67 ± 1337.44	6	PD2	4426.00 ± 435.29	6	PC5	4972.50 ± 530.01	6
AV4	3381.00 ± 587.07	6	PD3	2777.67 ± 464.10	6	PC6	3166.83 ± 374.56	6
AV5	4265.17 ± 665.29	6	PD4	2381.00 ± 288.30	6	ELG	8142.17 ± 509.46	6
L1	1857.83 ± 268.85	6	PL1	5997.67 ± 723.98	6			

*Underlined texts indicate missing/abnormal glomeruli*.

**Table 3:** Presence of anomalous missing ordinary glomeruli in the antennal lobe (AL) in the six males (M1–6) and females (F1–6) of *Plutella xylostella*.

	**M1**	**M2**	**M3**	**M4**	**M5**	**M6**		**F1**	**F2**	**F3**	**F4**	**F5**	**F6**
Total number	76	74	74	74	74	74	Total number	77	76	75	75	75	74
M5	+	+	+	–	+	+	AM4	+	+	+	+	+	–
V3	+	+	+	+	–	–	M3	+	+	–	–	+	–
PV2	+	–	–	–	–	+	M5	+	–	+	+	+	+
PV6	+	–	–	+	+	–	M6	+	+	–	–	–	–
							PL3	+	+	+	+	–	+

*+, presentation glomerulus in ALs. –, missing glomerulus in Als*.

### Landmark Glomeruli

There were several distinct glomeruli in both sexes which were used as landmarks for glomeruli identification and matching. For the male AL, in addition to the above-mentioned putative MGC, glomeruli AL2, PV1, PL2, AV3, AL4, PC1, PC4, and PD9 could be easily identified either by their marked locations, shapes or sizes. For example, the glomerulus AV3 was a flattened, elliptical sphere, located in the anterior-lateral region of the AL and under the MGC (Figure 5A). AL4 neighbored the MGC, and was larger than other ordinary glomeruli in this region (Figure 5A). PC1 was a slender cylinder, and located in the posterior-lateral region of the AL (Figure 5C). PC4 was a long elliptical sphere, located in the posterior-central region of the AL (Figure 5C). The larger glomerulus PD9 was located in the most posterior region of the AL, and close to the MGC (Figure 5C).

In the female ALs, in addition to the morphologically evident glomeruli ELG, AD3, and PV1, glomeruli AC2, AV5, PL5, and PL1 were also easily recognizable. AC2 was oblong in shape, and located in the most anterior region of the AL (Figure 8A). AV5 and PL5 were among the largest glomeruli in the ventral regions of the AL (Figures 8A,D). PL1 was the third largest glomerulus, and located in the posterior-central region of the AL (Figure 8C).

### Comparison of Male and Female ALs

Comparing the glomerular organization of the AL of male and female *P. xylostella* demonstrated both sexes possessed approximately the same number of AL glomeruli. Within the same sex, most glomeruli proved to be highly consistent in size, shape and position in the ALs. Nevertheless, a few anomalous glomeruli were missing in some ALs of both sexes (Table 3). Additionally, although the majority of glomeruli were between 1,000 and 3,000 μm^3^ in volumes in both sexes, there are some size distribution differences in the male and female glomeruli (Figure 9). The peak number of glomeruli in the males is with a volume between 1,000 and 2,000 μm^3^ while, in the females, the peak number of the glomeruli was with a volume between 2,000 and 3,000 μm^3^ (Figure 9).

Some special glomeruli were present in both sexes in the same position. For example, the largest glomeruli MGC in males and ELG in females occupy the same domain in both sexes. The second largest glomerulus PL2 in males and PL5 in females was situated under the MGC and ELG, respectively (Figures 5D, 8D; Tables 1, 2). The smallest glomerulus AL2 in males and AD3 in females was located in the anterior region of the AL and near to the entrance of the antennal nerve (Figures 5A, 8A; Tables 1, 2). Furthermore, PV1 was the only glomerulus not innervated by the antennal nerve, but by the labial palpus nerve in both sexes (Figure 10). This glomerulus was relatively large and located at the ventral side of the AL (Figures 5F, 8F). The glomerulus PV1 in the male was smaller than that in the females. Additionally, unlike other glomeruli in the AL which received neuronal input from ipsilateral antennal nerves, this glomeruli received innervation from both sides of the labial palpus nerve (Figure 10).

## Discussion

In this paper, we presented a complete 3-D reconstruction of the glomerular organization of *P. xylostella*, based on the systematic anatomical matching of glomeruli within and between the sexes. We found most glomeruli were apparently isomorphic between sexes. However, significant differences occurred in the presence, absence, sizes, or locations of some glomeruli.

### Number of Glomeruli in *P. xylostella*

Similar to other investigated insect species, the glomeruli are distributed around a central fiber core (Rospars and Hildebrand, 1992, 2000; Berg et al., 2002; Greiner et al., 2004). The number of glomeruli can vary greatly depending on the species (Rospars, 1988; Huetteroth and Schachtner, 2005); in Lepidoptera, the number of glomeruli ranges from 60 to 70 (Rospars, 1983; Rospars and Hildebrand, 1992, 2000; Berg et al., 2002; Sadek et al., 2002; Greiner et al., 2004; Masante-Roca et al., 2005; Namiki et al., 2014). The number of glomeruli found in *P. xylostella* is slightly higher than in the other moth species previously mentioned, which is not surprising because these species belong to very distantly related families. However, even closely related species have significant differences in the number of glomeruli. Generally, one glomerulus is the target site for one ORN type (Couto et al., 2005). The number of glomeruli is different in different insect species because they possess different lifestyles. When comparing two closely related species, *Cydia molesta* has fewer glomeruli than *Lobesia botrana*, and the former is an oligophagous insect species, while the latter is a polyphagous species; therefore, *C. molesta* may require a narrower range of olfactory cues to identify their hosts than that of *L. botrana* (Varela et al., 2009). When insects lack glomeruli caused by some factors, e.g., mutagenesis causes, these insects lose corresponding olfactory functions. *Orco* mutant ants (disrupted *orco*, a gene required for the function of all ORs) lack most of the ~500 glomeruli found in wild-type ants and are unable to perceive pheromones (Trible et al., 2017). Generally, insects with complex lifestyles (e.g., social insects) have relatively large numbers of glomeruli and use a large variety of chemical cues (Galizia et al., 1999; Kleineidam et al., 2005). *P. xylostella* are oligophagous insects (Renwick et al., 2006), and they have a relatively narrow range of olfactory cues. Thus, *P. xylostella* has lower numbers of glomeruli than those of the social insects studied to date. Nevertheless, comparing with other moths studied so far, *P. xylostella* shows a higher number of glomeruli suggesting a neuronal base for a more complex olfactory behavior.

### Macroglomeruli in the Male *P. xylostella*

MGC in the AL structure based on sex pheromone communication has been observed in a wide range of insects, such as cockroaches (Boeckh et al., 1987), bees (Brockmann and Brückner, 2001), ants (Nishikawa et al., 2008), fruit flies (Solari et al., 2016), and moths (Anton and Homberg, 1999; Hansson and Anton, 2000; Masante-Roca et al., 2005; Varela et al., 2009; Nirazawa et al., 2017). The MGC consists of 1–7 glomeruli situated closely together and is near the entrance of the antennal nerve in males (Hansson, 1995; Masante-Roca et al., 2005). In Lepidoptera, the number of MGC structures is different in different species, comprising one large glomerulus in *L. botrana* males (Masante-Roca et al., 2005), two large glomeruli in *M. brassicae* (Rospars, 1983), three glomeruli in *Helicoverpa assulta* males (Berg et al., 2002), *Manduca sexta* (Rospars and Hildebrand, 2000) and *Spodoptera littoralis* (Ochieng et al., 1995), and four glomeruli in *Agrotis ipsilon* (Greiner et al., 2004). We found the putative MGC contained three glomeruli in the males of *P. xylostella*. One of these glomeruli resembled the cumulus, which has been found in several other moths and processes sex pheromone information (Hansson and Anton, 2000; Masante-Roca et al., 2005; Varela et al., 2009; Nirazawa et al., 2017), while two other glomeruli, a and b, were positioned posteriorly and close to the cumulus.

The number of MGC structures has been reported correlated to the number of pheromone components in the respective species (Hansson et al., 1992; Ochieng et al., 1995; Berg et al., 1998; Varela et al., 2009). In *H. virescens*, the MGC contains four glomeruli and receptor neurons that respond to the major sex pheromone component (Z11–16:AL) projected to the large glomerulus cumulus, the second pheromone component (Z9-14:AL) projected to the dorso-medial glomerulus, and the interspecific signals (Z11-16:AC and Z11-16:OH) projected to two ventrally located glomeruli (Berg et al., 1998). Intriguingly, the sex pheromone *P. xylostella* comprises 3 components [(Z)-11-hexadecenal (Z11-16:Ald), (Z)-11-hexadecenyl acetate (Z11-16:OAc) and (Z)-11-hexadecen-1-ol (Z11-16:OH)] (Bignon et al., 2010). Z11-16:Ald and Z11-16:OAc are essential for attraction of male moths, Z11-16:OH has a synergistic effect on the two components (Koshihara and Yamada, 1980). Z11-16:Ald and Z11-16:OAc with a 10:90 blend could highly attract male *P. xylostella*, and addition of 1 or 10% of Z 11-16:OH to the blend significantly increased attraction (Bignon et al., 2010). Although the putative MGC contains 3 compartments which match the number of the pheromone components, the projection pattern of receptor neurons tuned to behaviorally active components in *P. xylostella* has not been studied. We will investigate the structure-function relationships in ORNs with a combination of electrophysiological and anatomical studies of the pheromone-specific ORNs.

### Enlarged Glomeruli in the Female *P. xylostella*

In the female *P. xylostella* the largest glomerulus, ELG, was found at the entrance of the antennal nerve, a location similar to the one found in males MGC in the antennal lobe. However, this glomerulus was not a complex structure and was much smaller than that of the male MGC. This morphologically distinct glomerulus is not universally present in the female AL. It has previously been described in some female moths, such as *Bombyx mori* (Koontz and Schneider, 1987), *M. sexta* (Rospars and Hildebrand, 2000), *H. virescens* (Berg et al., 2002) but not in *S. littoralis* (Ochieng et al., 1995), and *C. molesta* (Varela et al., 2009). Physiological characterization of a PN innervating this glomerulus showed that it processes a self-released sex pheromone in addition to plant volatiles (Ljungberg et al., 1993; Reisenman et al., 2004; Trona et al., 2010). The similarity of the ELG suggests a similar role. It remains to be clarified in *P. xylostella*.

### Other Glomeruli Between the Sexes

Male moths must locate a mating partner via the dedicated sex pheromones cues. Mated females must identify the suitable oviposition sites via plant volatiles. Therefore, it is not surprising the glomerular structure differs between the sexes. The specific ordinary glomeruli AL2 and PL2 were found close to the MGC. Due to the closeness with the putative MGC of these specific glomeruli, we speculate that these glomeruli might receive and process information regarding the female sex pheromone. In the female ALs, the specific ordinary glomeruli AD3 and PL5 were located near the ELG, and might be responsible for receiving/processing certain female specific behaviors-relative chemical cues (e.g., plant volatiles). More functional studies of these specific ordinary glomeruli are required to elucidate their true functions.

Glomerulus PV1 did not receive projections of the antennal ORNs but received input from the labial palpus nerve, and these glomeruli were located at the ventral of the AL in both sexes and assumed to be the labial pit organ glomerulus (LPOG). The LPOG is found in some species, e.g., the moth *Rhodogastria* (Bogner et al., 1986), *Pieris rapae* (Lee and Altner, 1986), *M. sexta* (Kent et al., 1986), mosquitoes (Anton et al., 2003), *C. molesta* (Varela et al., 2009), and *Helicoverpa armigera* (Zhao et al., 2013, 2016). The LPOG is specialized in sensing CO_2_ (Bogner et al., 1986; Stange, 1992; Guerenstein and Hildebrand, 2008; Ning et al., 2016). Glomerulus PV1 in ALs of *P. xylostella* is believed to process information on CO_2_ levels in the environment. Further functional studies (either single sensillum recording or Ca imaging) are needed to verify if this is the case in this insect species.

In conclusion, we provided a 3-D reconstruction of the glomerular structure in the AL of *P. xylostella*. This result provides a foundation for further studying of the olfactory information processing in this important economical pest.

## Author Contributions

XS, CH, and XY conceived and designed the study. XY and ZW acquired and analyzed the data. XY, JX, and CD analyzed and interpreted the data. XY and XS wrote the manuscript. CH provided research funding.

### Conflict of Interest Statement

The authors declare that the research was conducted in the absence of any commercial or financial relationships that could be construed as a potential conflict of interest.

## Supplementary Material

Supplementary Video 1 Video of consecutive frontal sections of a male *Plutella xylostella* antennal lobe with Propidium Iodide and Neurobiotin labeling.

Supplementary Video 2 Three-dimensional reconstruction of a male antennal lobe of *Plutella xylostella*.

Supplementary Video 3 Three-dimensional reconstruction of a female antennal lobe of *Plutella xylostella*.
